# Phytochrome B regulates resource allocation in *Brassica rapa*

**DOI:** 10.1093/jxb/ery080

**Published:** 2018-03-03

**Authors:** Andrej A Arsovski, Joseph E Zemke, Benjamin D Haagen, Soo-Hyung Kim, Jennifer L Nemhauser

**Affiliations:** 1 Department of Biology, University of Washington, Seattle, WA, USA; 2 School of Environmental and Forest Sciences, University of Washington, Seattle, WA, USA

**Keywords:** *Brassicaceae*, climate change, phytochromes, resource allocation

## Abstract

Crop biomass and yield are tightly linked to how the light signaling network translates information about the environment into allocation of resources, including photosynthates. Once activated, the phytochrome (phy) class of photoreceptors signal and re-deploy carbon resources to alter growth, plant architecture, and reproductive timing. Most of the previous characterization of the light-modulated growth program has been performed in the reference plant *Arabidopsis thaliana*. Here, we use *Brassica rapa* as a crop model to test for conservation of the phytochrome–carbon network. In response to elevated levels of CO_2_, *B. rapa* seedlings showed increases in hypocotyl length, shoot and root fresh weight, and the number of lateral roots. All of these responses were dependent on nitrogen and polar auxin transport. In addition, we identified putative *B. rapa* orthologs of *PhyB* and isolated two nonsense alleles. *BrphyB* mutants had significantly decreased or absent CO_2_-stimulated growth responses. Mutant seedlings also showed misregulation of auxin-dependent genes and genes involved in chloroplast development. Adult mutant plants had reduced chlorophyll levels, photosynthetic rate, stomatal index, and seed yield. These findings support a recently proposed holistic role for phytochromes in regulating resource allocation, biomass production, and metabolic state in the developing plant.

## Teaser

Crop yield is sensitive to biomass allocation. Here, we show that resource status modulates biomass partitioning in *Brassica rapa*, and that this calculation depends on *PhyB.*

## Introduction

‘Functional equilibrium’ describes the metabolic balancing act whereby plants adjust the allocation of biomass between carbon-acquiring, photosynthetic shoots and nutrient-absorbing roots (Brouwer, 1963; Thornley, 1972; Iwasa and Roughgarden, 1984; Poorter *et al.*, 2012). The biomass that gets invested in harvestable yield of crops is heavily influenced by this calculation. Light and CO_2_ availability can limit photosynthetic productivity of aboveground tissue and thereby belowground carbon allocation (Rogers *et al.*, 1994; Curtis and Wang, 1998; De Graaff *et al.*, 2006; Litton *et al.*, 2007; Dieleman *et al.*, 2010). The availability of nitrogen to the roots plays a particularly significant role in constraining plant growth and crop yield worldwide (Epstein and Bloom, 2005; Hirel *et al.*, 2011; Alvarez *et al.*, 2012). Numerous species respond to changes in nitrogen availability by altering the root/shoot biomass ratio to maintain nutrient balance (Aerts *et al.*, 1992; Scheible *et al.*, 1997; Hermans *et al.*, 2006; Grechi *et al.*, 2007; Li *et al.*, 2012).

The way in which plants respond to carbon and nitrogen supply is of particular interest in the context of increasing global atmospheric CO_2_ levels. Since the Industrial Revolution, atmospheric CO_2_ levels have increased from 280 ppm to >400 ppm at the time of writing, and are predicted to reach 730–1000 ppm by the end of the century (Intergovernmental Panel on Climate Change, 2007, 2014). CO_2_ directly affects plants through impacts on photosynthetic gas exchange and downstream developmental processes (Ainsworth and Long, 2005; Gray and Brady, 2016). In a long-term field experiment, elevated CO_2_ stimulated photosynthetic carbon assimilation rates by an average of ~30% across 40 species (Ainsworth and Long, 2005). A CO_2_-driven increase in shoot biomass has been shown to lead to significant increases in seed yield in many crops, including soybean, rice, bean, wheat, and peanut (Hatfield *et al.*, 2011). At the same time, increased carbon assimilation leads to higher nitrogen demand and often increased root growth. When carbon levels are increased by treatment with sugars or elevated CO_2_ in laboratory conditions, *Arabidopsis thaliana* seedlings produce more lateral roots (MacGregor *et al.*, 2008; Lilley *et al.*, 2012; Hachiya *et al.*, 2014). Natural C_3_–C_4_ grassland exposed to elevated atmospheric CO_2_ shows significantly increased community root biomass (Anderson *et al.*, 2010), and root biomass has been observed to increase significantly in response to elevated CO_2_ in many crops (Madhu and Hatfield, 2013).

Biomass allocation calculations can vary dramatically by species and environment. In a meta-analysis of CO_2_ responses, it was close to a 60:40 split between species that decreased versus increased their shoot/root ratio (Rogers *et al.*, 1995). The progressive nitrogen limitation hypothesis posits that nitrogen additions should enhance CO_2_ effects on plant productivity (Luo *et al.*, 2004). Nitrogen scarcity may therefore limit ecosystem response to elevated CO_2_ concentration (Oren *et al.*, 2001; Hungate *et al.*, 2003; Luo *et al.*, 2004; Reich *et al.*, 2006; Langley and Megonigal, 2010). Studies in *Brassica* show that yield improvements could be achieved with elevated CO_2_ but only with increased nitrogen supplementation (Uprety and Mahalaxmi, 2000). Similarly, elevated CO_2_ stimulates aboveground biomass in a grassland by up to 33%, but is dependent on water and nitrogen availability, with lower biomass stimulation observed in drier, lower nutrient conditions (Reich *et al.*, 2014). Understanding the molecular targets of elevated CO_2_, in addition to knowledge of how these targets impact biomass allocation, could provide guidance for crop breeding and management practices to optimize future yields.

Light signaling via photoreceptors acts at the intersection of plant growth control and metabolic homeostasis. The phytochrome family of photoreceptors responds primarily to red and far-red wavelengths, switching between Pr (inactive) and Pfr (active) isomeric forms (Fraser *et al.*, 2016). Plants with reduced *phyB* function have altered leaf area and slower growth compared with wild-type plants (Halliday *et al.*, 2003). One possible explanation for this retarded growth is reduced chlorophyll and, most probably, photosynthetic rate (Strasser *et al.*, 2010; Hu *et al.*, 2013). Phytochromes control chloroplast gene expression during photomorphogenesis as well as nuclear-encoded factors involved in chloroplast development (Waters *et al.*, 2008, 2009; Oh and Montgomery, 2014). Phytochrome B is also required for the light-dependent development of stomata (Casson *et al.*, 2009; Casson and Hetherington, 2014). Adult *phy* mutants in Arabidopsis have reduced CO_2_ uptake and sizeable reductions in overall growth (Yang *et al.*, 2016). In addition, phytochrome loss impacts core metabolism, notably the stress metabolites proline and raffinose. Mutants appear to be diverting resources from biomass production to improve resilience.

In this study, we tested the extent of PhyB’s effect on plants’ response to resource availability. We focused on *Brassica rapa*, as the *Brassica* genus includes species of worldwide economic importance, and their relatively recent divergence from Arabidopsis increased the likelihood of shared molecular pathways (Huang *et al.*, 2016). We established that a number of growth parameters are significantly affected by elevated CO_2_, including increased hypocotyl length, shoot and root fresh weight, and the number of lateral roots. All of these responses required adequate nitrogen and polar auxin transport, as has been observed in Arabidopsis. We also identified *B. rapa* orthologs of *PhyB* and found that loss of *BrPhyB* function abrogated seedling responses to high CO_2_. Moreover, adult mutants had reductions in chlorophyll, photosynthetic rate, and stomatal index. Together, these results highlight the role of phytochrome photoreceptors in shaping plant architecture and biomass partitioning across the plant life cycle.

## Materials and methods

### Plant materials and growth conditions

#### Seedlings

**Fig. 1.**
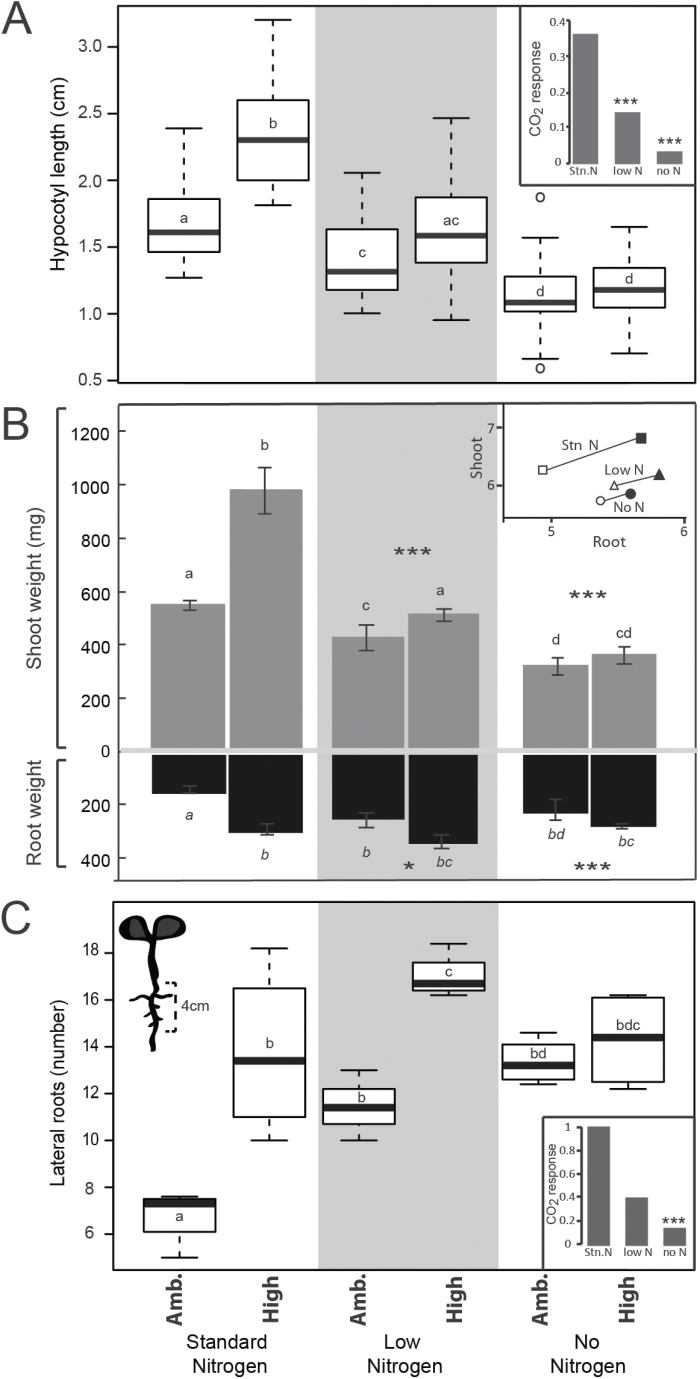
Nitrogen availability limits growth increase in high CO_2_. (A) Average hypocotyl lengths of 10-day-old *B. rapa* seedlings on media containing standard, low, or no nitrogen in ambient (400 ppm) or high (1000 ppm) CO_2_. The bold line is the median of at least four independent biological replicates with five seedlings per replicate. (B) Average shoot and root weights of the same seedlings from (A). Inset shows ln(root fresh weight, mg) on the *x*-axis and ln(shoot fresh weight, mg) on the *y*-axis, Stn N=standard media, low N=low nitrogen medium, No N=no nitrogen medium. Filled marker points indicate high CO_2_; open marker points are ambient CO_2_. (C) Number of lateral roots within the 4 cm of the primary root near the crown of seedlings from (A), counted on day 7. Box plot ‘whiskers’ denote the maximum and minimum of the data, circles are outliers, the top edge of the box is the third quartile of the data, and the bottom edge is the first quartile. Different letters indicate that samples had significantly different means (ANOVA and Tukey HSD multiple comparison test). Insets show fold response to high CO_2_ compared with ambient CO_2_. Asterisks indicate which conditions give a significantly different response to CO_2_ when compared with standard conditions (***P<0.001, *P<0.05, ANOVA).

**Fig. 2.**
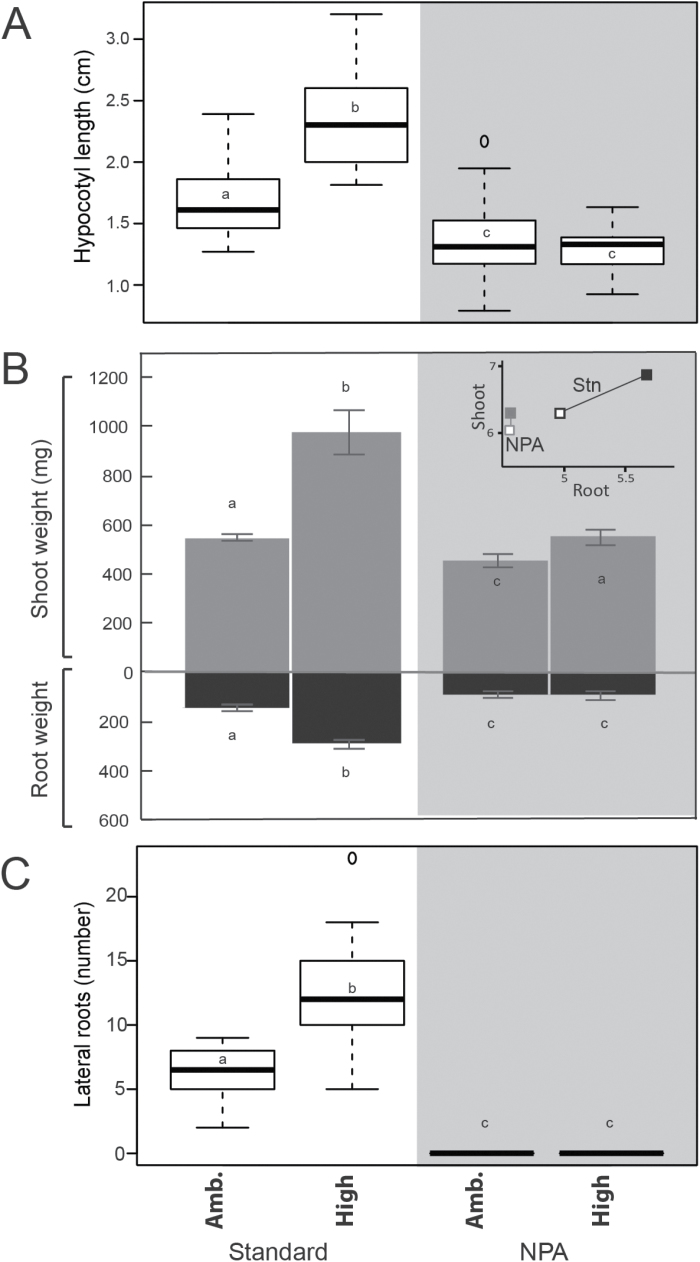
CO_2_-responsive growth requires auxin transport. (A) Average hypocotyl lengths of 10-day-old *B. rapa* seedlings on standard media with mock or NPA treatment, in ambient (400 ppm) or high (1000 ppm) CO_2_. The bold black line is the median of at least four independent biological replicates with five seedlings per replicate. (B) Average shoot and root weights of the same seedlings from (A). Inset shows ln(root fresh weight, mg) on the *x*-axis and ln(shoot fresh weight, mg) on the *y*-axis, Stn=standard medium, NPA= NPA medium. Filled marker points indicate high CO_2_; open marker points are ambient CO_2_. (C) Number of lateral roots within the 4 cm of primary root near the crown of seedlings from (A), counted on day 7. Letters indicate significant differences of pairwise mean comparisons (ANOVA and Tukey HSD multiple comparison test). Seedlings exposed to NPA have a significantly reduced response to elevated CO_2_ (*P*<0.01, ANOVA).

**Fig. 3.**
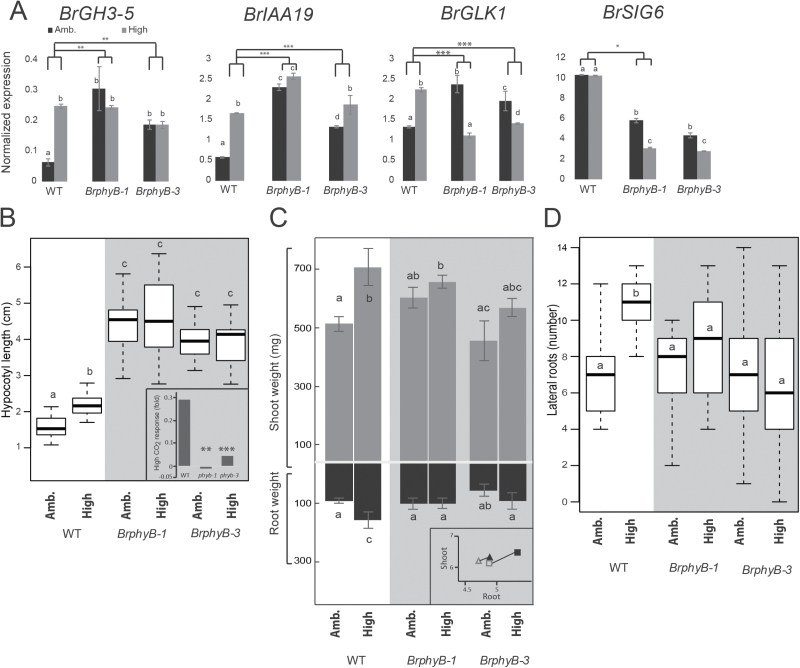
Response to elevated CO_2_ requires *BrPhyB*. (A) Gene expression (qRT-PCR) in *phyB* mutants. Expression relative to *PP2A* control. Different letters denote statistical significance compared with the wild type (*P*<0.001, ANOVA and Tukey HSD multiple comparison test). Asterisks indicate a significantly different response to CO_2_ compared with the wild type (***P<0.001, **P<0.01, *P<0.05, ANOVA, *n*=3). (B) Average hypocotyl lengths of 10-day-old *B. rapa* seedlings on standard, low, or no nitrogen media in ambient (400 ppm) or high (800 ppm) CO_2_. The bold black line is the median of at least four independent biological replicates with five seedlings per replicate. The inset shows the fold response to high CO_2_ compared with ambient CO_2_. Asterisks indicate that all mutants have a significantly different response to CO_2_ than the wild type (***P<0.001, **P<0.01, *P<0.05, ANOVA). (C) Average shoot and root weights of the same seedlings from (A); inset shows ln(root fresh weight, mg) on the *x*-axis and ln(shoot fresh weight on the *y*-axis, square= wild type, triangle=average of *phyB-1* and *phyB-3*. Filled marker points indicate high CO_2_; open marker points are ambient CO_2_. CO_2_ response in all mutants is significantly different from that in the wild type (*P*<0.05, ANOVA). (D) Number of lateral roots within the 4 cm of primary root near the crown of seedlings from (A), counted on day 7. CO_2_ response in all mutants is significantly different from that of the wild type (*P*<0.001, ANOVA). Letters show significant differences of pairwise mean comparisons (ANOVA and Tukey HSD multiple comparison test).

The *B. rapa* wild-type R-o-18 and *phyB* mutant lines were obtained from the John Innes Centre’s RevGENUK resource (http://revgenuk.jic.ac.uk/) (Stephenson *et al.*, 2010). Seeds were sterilized with Cl_2_ gas for 4 h in a sealed bell jar (Clough and Bent, 1998), sown on plates, and stratified in the dark at 4 °C for 3 d. Standard medium was 0.5× Linsmaier and Skoog (LS) [LSP03, Caisson Laboratories, Inc.; N sources were 1650 mg l^–1^ NH_4_NO_3_ and 1900 mg l^–1^ KNO_3_] with 0.8% phytoagar (40100072-1, Plant Media: bioWORLD). Germinated seedlings were selected for similar developmental stage and moved to plates containing varying nitrogen levels or chemical treatment. For nitrogen-related experiments (Fig. 1; Supplementary Figs S1, S2 at *JXB* online), medium was prepared from 0.5× Murashige and Skoog without nitrogenous compounds (MSP07, Caisson Laboratories, Inc.) and supplemented with either 0.5 mM KNO_3_ (low N) or 0.5 mM KCl (no N). *N*-1-napthylphthalamic acid (NPA, 100 mM) was added directly to 0.5× LS medium prior to pouring (Fig. 2). All other seedling experiments used the 0.5× LS (standard) medium. Plates were placed vertically at dawn in a Reliance LED growth chamber (www.reliancelabs.com) set at 20 °C. LED intensities were set at 9.23% for 470 nm, 11.64% for 660 nm, 9% for 730 nm, and 5.67% for 447 nm. Light intensity was 100 µmol m^−2^ s^−1^ with short-day conditions (8 h light, 16 h dark). For CO_2_ experiments, ambient CO_2_ was set at 400 ppm for ambient (A) and 1000 ppm for high (H) CO_2_. Chamber data were logged every 5 min. A mechanical issue led to elevated CO_2_ levels being only 800 ppm in the experiments summarized in Fig. 3.

#### Adult plants

Seeds were sown directly onto our standard soil mix of 1:1 Sunshine Mix #4^®^ (SunGro Horticulture):vermiculite. Plants were grown four per 2.6 liter square pots (McConkey Grower Products; Sumner, WA, USA). Plants were bottom-watered daily in long-day conditions Percival E-30B growth chamber set at 20 °C (16 h light, 8 h dark; ~115 µmol m^−2^ s^−1^) for 3 weeks.

### Plant measurements

#### Seedlings

Hypocotyl lengths and lateral root number were measured from scans of vertical plates using ImageJ software (http:/rsb.info.nih.gov/ij/) in at least four independent experiments of five seedlings. Plates were scanned daily from day 4 to day 10 for time course experiments. Lateral roots were counted in the first 4 cm of the primary root at day 7. Lateral roots were counted on day 7 to avoid lateral root entanglement between adjacent seedlings. At this stage, essentially all emerged lateral roots are found within the first 4 cm of the primary root. Hypocotyl lengths were measured at day 10. Biomass data were collected by harvesting immediately after the final scan on day 10. Five seedlings from each replicate plate were dissected, pooled, and weighed using an analytical balance, so that each biological replicate of five seedlings represents one fresh or dry weight data point. Fresh weight was recorded immediately; tissue was then dried at 70 °C for 4 d and measured again to determine dry weight.

#### Adult plants

For chlorophyll, ethanol extractions were done as in Yang *et al.* (2016). Determinations were done by measuring optical density at 645 nm and 665 nm using an Epoch Microplate Spectrophotometer (www.biotek.com). Chl *a*=5.21_*A*665_−2.07_*A*645_; Chl *b*=9.29_*A*645_−2.74_*A*665_. Values were combined for the ‘total chlorophyll’ value. Photosynthetic rate was measured using a portable LI-COR LI-6400XT Photosynthesis system (www.licor.com). Photosynthetic rate was measured every 5 min for a total of 15 min to allow for acclimation and plateau (light, 1500 µmol m^−2^ s^−1^; temperature, 20 °C; CO_2_, 450 ppm). The first or second true leaf was measured for all plants tested. For the stomatal index, epidermal peels were done by applying clear nail polish to the first or second true leaf of 3-week-old plants for 30–60 s. A strip of clear packing tape was then applied over the nail polish and promptly removed and placed on a microscope slide. Differential interference contrast (DIC) images were taken using a Leica DMI300B inverted microscope, and stomatal and pavement cells were counted manually using Image J software. For fresh and dry weight, the aboveground tissue of at least six plants per genotype were cut and weighed, dried at 70 °C for 7 d, and weighed again. For seed yield, at least six plants of each genotype were harvested and all viable seeds were manually counted.

### RNA extraction and quantitative real-time PCR (qRT-PCR) analysis

Expression analysis was performed on four whole seedlings per genotype collected at the end of the day on day 10. A 120 mg (FW) aliquot of each sample was immediately frozen in liquid nitrogen and stored at –80 °C until processing. Frozen tissue was ground in liquid nitrogen and total RNA was extracted using the GE Illustra RNA kit (GE Life Sciences), and 2 µg of eluted RNA was used for cDNA synthesis employing iScript (Bio-Rad). Samples were analyzed using SYBR Green Supermix (Bio-Rad) reactions run in a C100 Thermal Cycler (Bio-Rad) fitted with a CFX96 Real-Time Detection System (Bio-Rad). Relative expression levels were calculated using the formula (E_target_)^−CPtarget^/(E_ref_)^−CPref^ (Pfaffl, 2001) and normalized to the *B. rapa PP2A* (Brara.F00691) reference gene. qPCR primer sequences are as follows: *PP2A* (forward 5'-TCGGTGGTAACGCCCCCGAT-3'; reverse 5'-CGACTCTCGTGGTTCCCTCGC-3'); for *PHYB* (Brara.E02473) (forward 5'-GCGCTCAGAGGGGACGAGGA-3'; reverse 5'-GCGGTGTTCCACTCCAGGCA-3'); for *BrSIG6* (Brara.D02249) (forward 5'-ACGCTTGCCGGAGAACGTGTA-3'; reverse 5'-ACACCAACGTGGCCTGCGAG-3'); *BrGLK1* (Brara.I04696) (forward 5'-AAGCCACCCGCTGTTGACGG-3'; reverse 5'-GCCTCAGG CGCTTGTCCGTT-3'); BrGH3-5 (Brara.K00097) (forward 5'-GACCC TTACACCGACTACAC-3'; reverse 5'-CATAAGCCACAAAGCAT CTGAG-3'); BrIAA19 (Brara.C03574) (5'-TCTCGATAAGCTCTT CAGTTTCC-3'; reverse 5'-CAGTCTCCATCTTTGTCTTCGT-3').

### Sequence alignment and cluster analysis

A phylogenetic tree was constructed using Geneious Pro 5.4.6 Biomatters Ltd. and sequences obtained from Arabidopsis.org and Phytozome.net. Sequence alignment was done using Phytozome.net and ClustalW (Larkin *et al.*, 2007; Goodstein *et al.*, 2012).

## Results

### Carbon and nitrogen availability alter development and biomass allocation in *B. rapa*

Increased carbon availability in the form of sucrose or elevated CO_2_ stimulates seedling growth in *A. thaliana*, but only in the presence of sufficient nitrogen (Stewart Lilley *et al.*, 2013). To test whether this same relationship held true for *B. rapa*, we exposed seedlings to high carbon environments under three nitrogen conditions (Fig. 1; Supplementary Fig. S1). Seedlings were grown for 10 days on standard growth medium, limited nitrogen (called low hereafter), or no nitrogen in either 400 ppm (ambient) or 1000 ppm (high) CO_2_. High CO_2_ conditions increased hypocotyl length for seedlings grown on standard medium by 37% (Fig. 1A; Supplementary Fig. S1; *P* < 0.001). This response was eliminated when plants were grown in low or no nitrogen (Fig. 1A; Supplementary Fig. S1; *P* < 0.001).

We dissected the seedlings at the end of the experiment to measure shoot and root fresh weights. When grown on standard media, average fresh weights of both tissues increased ~2-fold in response to high levels of CO_2_, with a root/shoot ratio increase from 0.26 to 0.29 (Fig. 1B, inset). The increased aboveground biomass response was significantly reduced when seedlings were grown on media containing low nitrogen and eliminated in no nitrogen media (*P*<0.001 for both above- and belowground fresh weight). In low nitrogen conditions, root/shoot ratios increased to 0.57 in ambient conditions and 0.65 in response to elevated CO_2_. The biomass difference between above- and belowground tissue was further reduced in the no nitrogen conditions (0.68–0.75). Dry weights showed similar trends (Supplementary Fig. S2).

Limiting nitrogen stimulated root fresh weight in ambient CO_2_. Root architecture was also affected (Supplementary Fig. S1). To determine if root branching was significantly increased, we counted the number of emerged lateral roots within the first 4 cm of the crown in 7-day-old seedlings (Fig. 1C). Nitrogen limitation in ambient conditions increased lateral root number to a similar degree as high CO_2_ (~2-fold more lateral roots in both low or no nitrogen compared with standard medium, *P*<0.001). High CO_2_ led to a somewhat diminished response in low nitrogen conditions (1.4-fold more lateral roots than in ambient conditions) and a lack of response when nitrogen was left out of the media. These results suggest that carbon and nitrogen availability co-ordinately shape *B. rapa* root architecture.

### Carbon-induced growth requires polar auxin transport

A number of lines of evidence demonstrate a link between sucrose and auxin in carbon-induced growth. Auxin biosynthesis and response genes are up-regulated by sugar treatment (Mishra *et al.*, 2009; Lilley *et al.*, 2012; Sairanen *et al.*, 2012). Similarly, Arabidopsis seedlings grown under elevated CO_2_ exhibit an increase of indole acetic acid (IAA) concentration in the shoots (Hachiya *et al.*, 2014). Both sucrose and auxin alter the dynamics of *A. thaliana* seedling growth by amplifying the rate and duration of hypocotyl elongation (Stewart *et al.*, 2011). To determine whether the increased hypocotyl length observed when *B. rapa* seedlings were grown in high CO_2_ required auxin, we grew seedlings on medium containing NPA, an inhibitor of auxin transport from the shoot to the root. NPA treatment completely abolished CO_2_-induced growth (Fig. 2A). This effect was mirrored in fresh weights. Whereas seedlings grown on standard medium had a root to shoot increase in response to high CO_2_, seedlings exposed to NPA had a decreased root/shoot ratio (0.19 to 0.17; Fig. 2B, inset, *P*<0.001). Consistent with this trend, lateral root growth was also completely suppressed by NPA treatment (Fig. 2C).

If high CO_2_ stimulates an auxin increase, then we would expect to see a concomitant induction of early auxin-responsive genes. To test this prediction, we assayed the expression of two *B. rapa* genes known to be up-regulated in response to exogenous auxin treatment (Procko *et al.*, 2014). *BrIAA19* and *BrGH3-5* are the likely orthologs of the Arabidopsis auxin co-repressor *INDOLE-3-ACETIC ACID INDUCIBLE19* and the IAA-amido synthase *GRETCHEN HAGEN 3-5*, respectively. Indeed, *BrGH3-5* and *BrIAA19* expression was significantly higher in seedlings grown in high CO_2_ when compared with those grown in ambient conditions (Fig. 3A).

### PhyB function is conserved across the *Brassicaceae*

*Phy* genes are central regulators of carbon allocation (Yang *et al.*, 2016). Here, we identified putative *Phy B. rapa* orthologs from analysis of the completed *B. rapa* genome (Lagercrantz and Lydiate, 1996; Trick *et al.*, 2009; Wang *et al.*, 2011). Using Phytozyme (Goodstein *et al.*, 2012), we queried all Arabidopsis *Phy* genes for possible *B. rapa* orthologs, in addition to other sequenced members of the *Brassicaceae*. *Brassica rapa* shares two paleotetraploidy events (beta and alpha) with Arabidopsis, and additionally underwent a whole-genome triplication thought to have occurred between 13 and 17 million years ago (Yang *et al.*, 1999; Town *et al.*, 2006; Beilstein *et al.*, 2010). The recent sequencing of the *B. rapa* genome confirmed the almost complete triplication of the genome compared with Arabidopsis and found that genes that underlie environmental adaptability are over-retained in *B. rapa* (Wang *et al.*, 2011).

A phylogenetic analysis of putative *Phy* genes across the sequenced *Brassicaceae* showed strong conservation among the five Phy clades represented by *PhyA–PhyE* in Arabidopsis (Supplementary Fig. S3). Within the *B. rapa* genome, only *PhyA* appears to have retained a duplicate homolog. There is probably only one *PhyB* ortholog and no likely ortholog for the closely related *AtPhyD*. Sequence alignment of Brara.E02473 to Arabidopsis *PhyB* and other *Brassicaceae* orthologs shows high conservation of the GAF, phytochrome and histidine kinase related domain (HKRD) (Supplementary Fig. S4). A mutant screen of the Wisconsin Fast Plant (WFP) self-compatible *B. rapa* variety also identified Brara.E02473 as a putative Arabidopsis *PhyB* ortholog (Procko *et al.*, 2014)

To test for functional conservation, we obtained multiple mutant alleles for *BrPhyB* (Brara.E02473) (Supplementary Fig. S4). The *BrphyB-1* and *BrphyB-3* alleles have nonsense mutations at amino acids 396 and 407, respectively (Supplementary Fig. S3). When grown in red light, seedlings with either mutation exhibited a nearly identical stereotypical *phyB* mutant phenotype with highly elongated hypocotyls and small, closed cotyledons (Supplementary Fig. S5A). Both mutants had an ~5-fold decrease in *BrPhyB* transcript level compared with wild-type seedlings (Supplementary Fig. S5B).

### BrPhyB is involved in resource allocation

Phytochromes are master regulators in organismal responses to environmental signals, controlling distinct aspects of nuclear and chloroplast gene expression (Chun *et al.*, 2001; Thum *et al.*, 2001; Oh and Montgomery, 2014). To examine whether transcriptional responses were affected in *BrphyB* mutant seedlings, we assayed the expression of genes known to be regulated by BrPhyB or involved in the high CO_2_ response. In *BrphyB-1* and *BrphyB-3* mutant seedlings grown in ambient CO_2_, transcript levels of *BrGH3-5* and *BrIAA19* were significantly higher than in wild-type seedlings; moreover, growth in high CO_2_ had no effect on expression of either gene (Fig. 3A, *P*<0.01 and *P*<0.001, response to high CO_2_ compared with wild-type seedlings, respectively, ANOVA). In Arabidopsis, *GOLDEN2-LIKE 1* (*GLK1*) affects the expression of nuclear photosynthetic genes involved in chloroplast development (Waters *et al.*, 2008, 2009) and its expression is significantly reduced in *phyAphyB* double mutants (Oh and Montgomery, 2014). Similarly, the chloroplast-targeted transcriptional regulator *SIG6* is involved in chlorophyll accumulation and plastid development (Kanamaru *et al.*, 2001; Ishizaki *et al.*, 2005), and is regulated by PhyB (Oh and Montgomery, 2014). We identified likely orthologs of GLK1 and SIG6 in *B. rapa*, each of which appears to be represented by a single gene. In *B. rapa* wild-type seedlings, *BrGLK1* expression increases 70% in response to high CO_2_. In *BrphyB-1* and *BrphyB-3* seedlings, expression is significantly higher in ambient CO_2_ compared with wild-type seedlings and decreases in response to high CO_2_ (Fig. 3A, *P*<0.001, ANOVA). CO_2_ levels do not affect *BrSIG6* expression in wild-type plants; however, mutations in *BrPhyB* seedlings significantly decrease expression of *BrSIG6* and lead to a negative response to elevated CO_2_ conditions (Fig. 3A, *P*<0.05 for *BrphyB-1*, ANOVA).

If the *Phy* genes are involved in the carbon-sensing network, as suggested by work in Arabidopsis (Lilley *et al.*, 2012; Yang *et al.*, 2016), then *B. rapa BrphyB* mutants should show a reduced seedling response to elevated CO_2_. To test this hypothesis, we grew wild-type and *BrphyB* seedlings in ambient and elevated CO_2_ conditions (Fig. 3; Supplementary Fig. S6). In response to elevated CO_2_, wild-type seedlings had a nearly 40% increase in average hypocotyl length. While significantly longer in ambient CO_2_ than those of the wild type, hypocotyls of *BrphyB-1* and *BrphyB-3* showed essentially no response to elevated CO_2_ (Fig. 3B; Supplementary Fig. S6*P*<0.01 and *P*<0.001, respectively).

Other growth impacts of high CO_2_ mirrored the hypocotyl results. Wild-type seedlings had an ~40% increase in shoot fresh weight when grown in elevated CO_2_ compared with growth in ambient conditions (Fig. 3C). In comparison, increased CO_2_ had essentially no impact on *BrphyB-1* and *BrphyB-3* shoot biomass (Fig. 3C; *P*<0.01, ANOVA). As a result, root/shoot ratios were significantly altered in both alleles (Fig. 3C, inset). Carbon effects on root architecture were also dependent on *BrPhyB.* The number of lateral roots in *BrphyB* mutants varied widely among individuals (particularly in *BrphyB-3*), but the average number did not significantly increase in response to high CO_2_ (Fig. 3D, *P*<0.001 for all three alleles, ANOVA).

### BrPhyB is required for normal photosynthetic function and seed yield

**Fig. 4.**
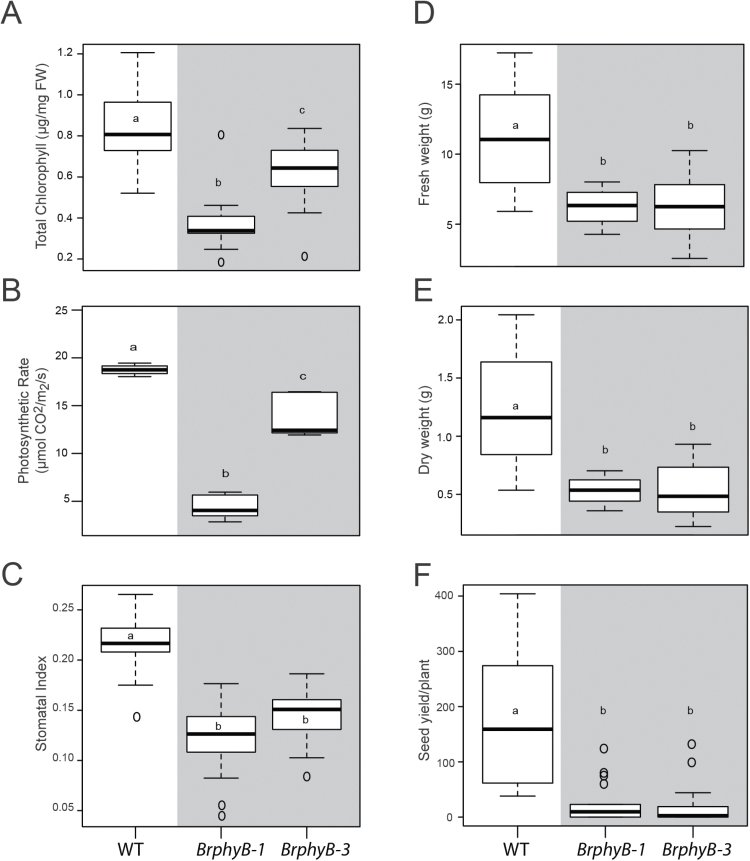
BrPHYB is required for a normal photosynthetic rate. (A) Total chlorophyll (ChlA+ChlB) in leaf tissue from at least twelve, 3-week-old wild-type and *phyB* mutant plants. Measured as µg mg^–1^ of harvested fresh tissue. (B) Photosynthetic rate (µmol CO_2_ m^–2^ s^–1^) from the same plants as (A). (C) Stomatal index measured as the number of stomata/number of epidermal cells from the same plants as (A). (D) Fresh weight of aboveground tissue from at least five, 6-week-old plants. (E) Dry weight of 6-week-old plants from (E). (F) Seed yield at harvest. Letters show significant differences of pairwise mean comparisons (ANOVA and Tukey HSD multiple comparison test).

Red light stimulates photosynthetic pigment production, and mutants with reduced phy function have significantly lower chlorophyll levels in Arabidopsis (Ghassemian *et al.*, 2006; Strasser *et al.*, 2010; Hu *et al.*, 2013). In addition, adult *phy* mutants have reduced CO_2_ uptake and significant reductions in overall growth (Yang *et al.*, 2016). At 3 weeks old, *BrphyB-1* and *BrphyB-3* mutant plants are noticeably paler compared with their wild-type counterparts. Predictably, total chlorophyll is significantly reduced in both mutant alleles (Fig. 4A, ANOVA and Tukey HSD). Not surprisingly, photosynthetic rates measured at ambient CO_2_ levels and a high light level are also significantly reduced in mutant plants (Fig. 4B; Supplementary Fig. S7, ANOVA and Tukey HSD).

Stomata are pores found on the surfaces of leaves. They regulate gas exchange, and are another component of photosynthetic productivity that is regulated by photoreceptors (Boccalandro *et al.*, 2009; Casson *et al.*, 2009; Kang *et al.*, 2009). PhyB, specifically, is required for light-mediated systemic control of stomatal development (Casson and Hetherington, 2014). In another indication of conservation of photoreceptor function between Arabidopsis and *B. rapa*, the stomatal index was significantly reduced in *BrphyB-1* and *BrphyB-3* mutant plants compared with the wild type (Fig. 4C).

Adult *BrphyB* mutant plants also had significant reductions in weight and seed yield. Both *phyB* alleles had a 42% decrease in fresh weight compared with wild-type plants. Six-week-old *BrphyB-1* and *BrphyB-3* plants had a 53% and 55% reduction in dry weight compared with wild-type plants, respectively (Fig. 4D, E; ANOVA and Tukey HSD). These are similar effects to what has been observed in Arabidopsis phytochrome mutants (Halliday *et al.*, 2003; Yang *et al.*, 2016). In addition, *BrphyB* mutant plants had significant reductions in total seed yield. *BrphyB-1* and *BrphyB-3* plants had an 87% and 90% reduction in the number of seeds per plant, respectively, when compared with wild-type plants (Fig. 4F ANOVA and Tukey HSD).

## Discussion

Climate change and the resultant shifts in temperature, atmospheric composition, and precipitation present a critical challenge for plant life on Earth. Potential adaptive responses can take the form of altered initiation and/or timing of developmental events, and changes in the final form or architecture of organs and whole plants. How individual crop species respond to new and potentially more variable conditions will dramatically impact crop yield and global food security. In this work, we have shown that the agriculturally important species *B. rapa* demonstrates shifts in biomass allocation in response to CO_2_ and nitrogen availability (Fig. 1). When nitrogen is not limiting, an increase in CO_2_ leads to increased growth, in both shoots and roots. However, when CO_2_ levels are high but nitrogen is in short supply, the seedling allocates resources to roots at the expense of shoots (Fig. 1; Supplementary Fig. S1). The responses we observed are in line with those seen for other species in field conditions (Ainsworth and Long, 2005; Reich *et al.*, 2014), highlighting the value of adding *B. rapa* as a laboratory crop model for studying the molecular mechanisms underlying functional equilibrium.

Phys have recently been proposed as regulators of carbon supply, metabolic status, and biomass production in Arabidopsis (Yang *et al.*, 2016). Our results extend this model to *B. rapa*. We identified two mutant alleles of the *B. rapa PhyB* ortholog. Compared with wild-type plants, hypocotyls of *BrphyB* mutants were longer in ambient CO_2_, but showed impaired high CO_2_-induced elongation (Fig. 3B). This pattern is consistent with previously described phenotypes in Arabidopsis. The inability to respond to carbon availability may be attributed to other phytochrome mutant phenotypes reported in Arabidopsis and also observed here in *B. rapa* (Fig. 4), such as reduced chlorophyll levels (Ghassemian *et al.*, 2006; Strasser *et al.*, 2010; Hu *et al.*, 2013; Yang *et al.*, 2016) or a decreased stomatal ratio (Boccalandro *et al.*, 2009). Mature *BrphyB* plants had reduced chlorophyll content, stomatal index, as well as photosynthetic rate when measured at high light levels (Fig. 4). Ectopic expression of Arabidopsis *PhyB* increases tuber yield, and cotton plant growth and yield at low light levels only (Schittenhelm *et al.*, 2004; Rao *et al.*, 2011), suggesting that the decrease in photosynthetic rate observed here may not be relevant in field conditions. However, the combined effect of chlorophyll levels, stomatal index, and auxin signaling—all affected by the *phyB* mutation in *B. rapa* and potentially working in concert*—*probably impact the whole plant response to high atmospheric CO_2_.

Detailed analysis of our *BrphyB* mutants across developmental time will be needed to elucidate the full extent of conservation of the Phy–carbon network across the *Brassicaceae* and into other plants of interest. Such work can lead to better models for plant growth that will facilitate improved predictions of how plants will respond to future climate conditions and can guide the selection of targets for engineering.

## Supplementary data

Supplementary data are available at *JXB* online.

Fig. S1. Carbon and nitrogen availability shape seedling architecture.

Fig. S2. Dry weight of 10-day-old seedlings on variable nitrogen media.

Fig. S3. Amino acid alignment of *Phy* homologs in the *Brassicaceae*.

Fig. S4. Amino acid alignment of putative *Brasicaceae PhyB* orthologs.

Fig. S5. (A) *PhyB* expression in *B. rapa* mutants. (B) Wild-type and *BrphyB* mutant seedlings grown under red light for 4 d.

Fig. S6. Response to elevated CO_2_ requires *PhyB*.

Fig. S7. Photosynthetic and stomatal conductance time course of 3-week-old *B.rapa* wild-type plants

Supplementary Figures

## Abbreviations:

NPA: 1-*N*-napthylphthalamic acid
phy: phytochrome.
