# Supplementary material for: Phytochrome B regulates resource allocation in Brassica rapa
Source: J Exp Bot. 2018 Mar 3;69(11):2837–46. doi: 10.1093/jxb/ery080 (PMC5961229; doi:10.1093/jxb/ery080)
Supplement: Supplementary Figures [file ery080_suppl_supplementary-figures.pdf]

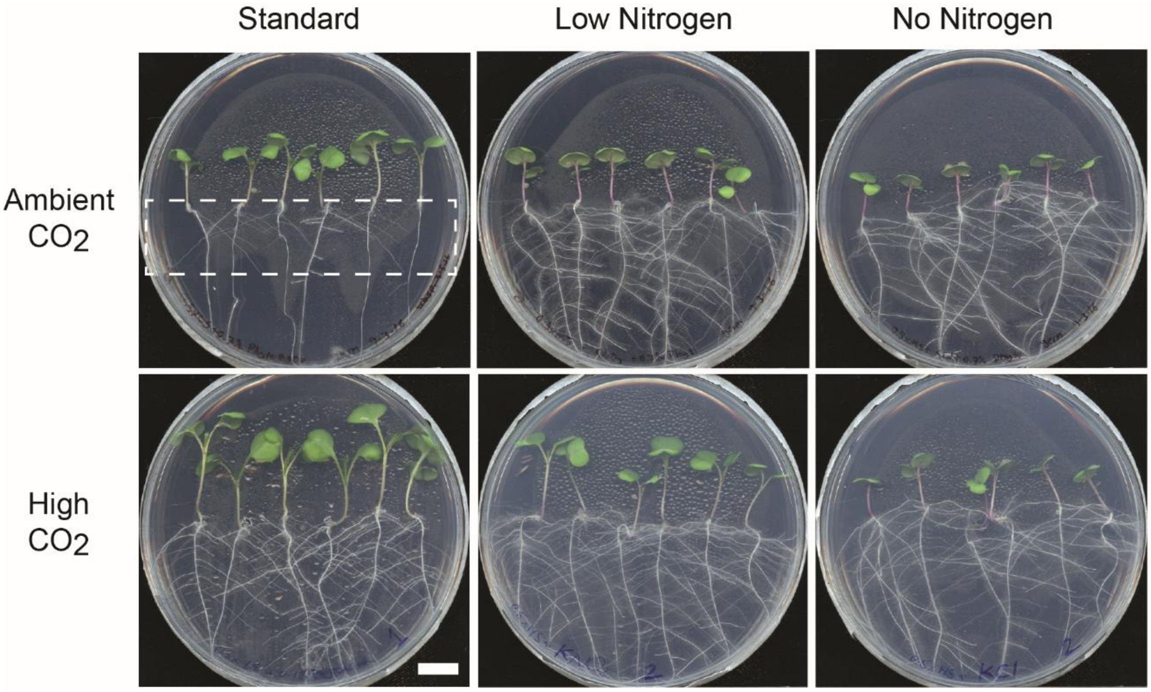

Supplemental Figure 1: 10 day old *B.rapa* seedlings have different shoot and root growth dynamics in response to carbon and nitrogen availability. Scale bar is 1cm, dashed rectangle indicates area where lateral roots were counted.

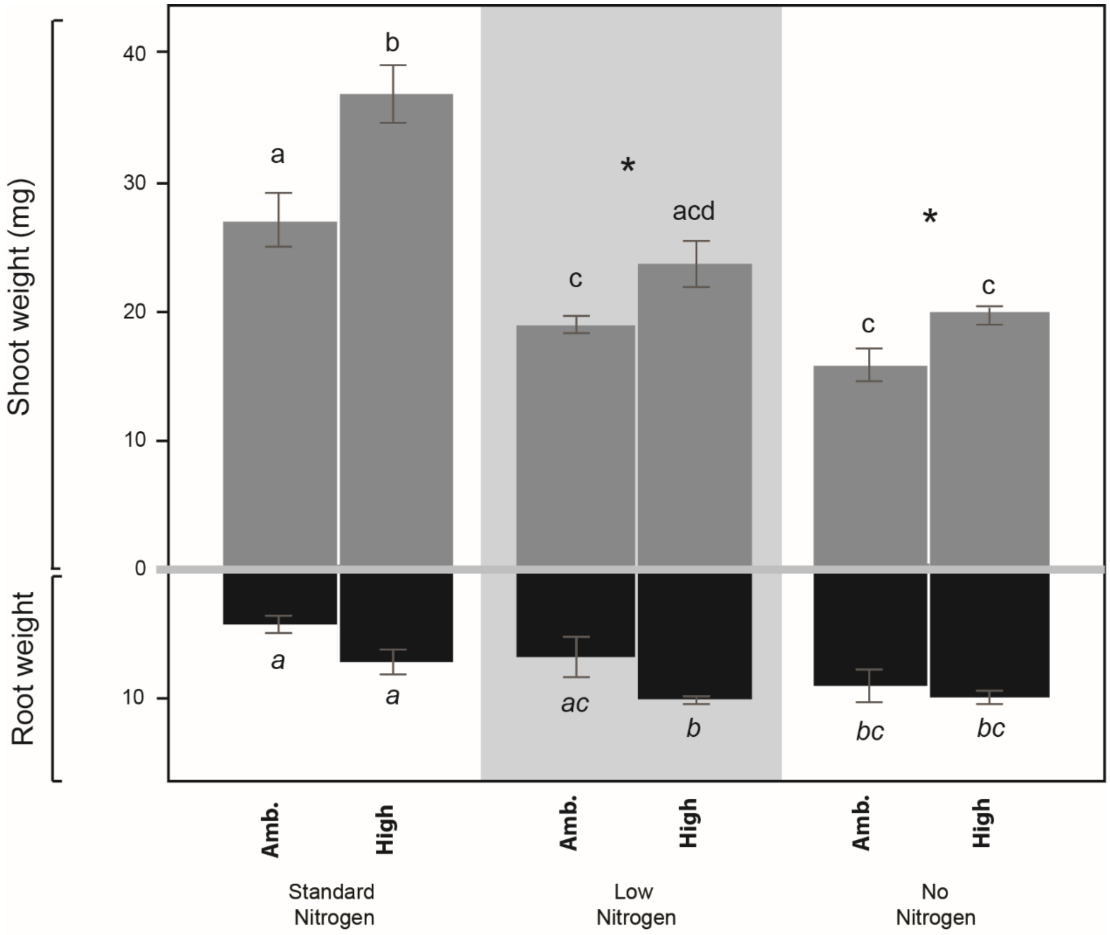

Supplemental Figure 2: 10 day seedling dry weight.

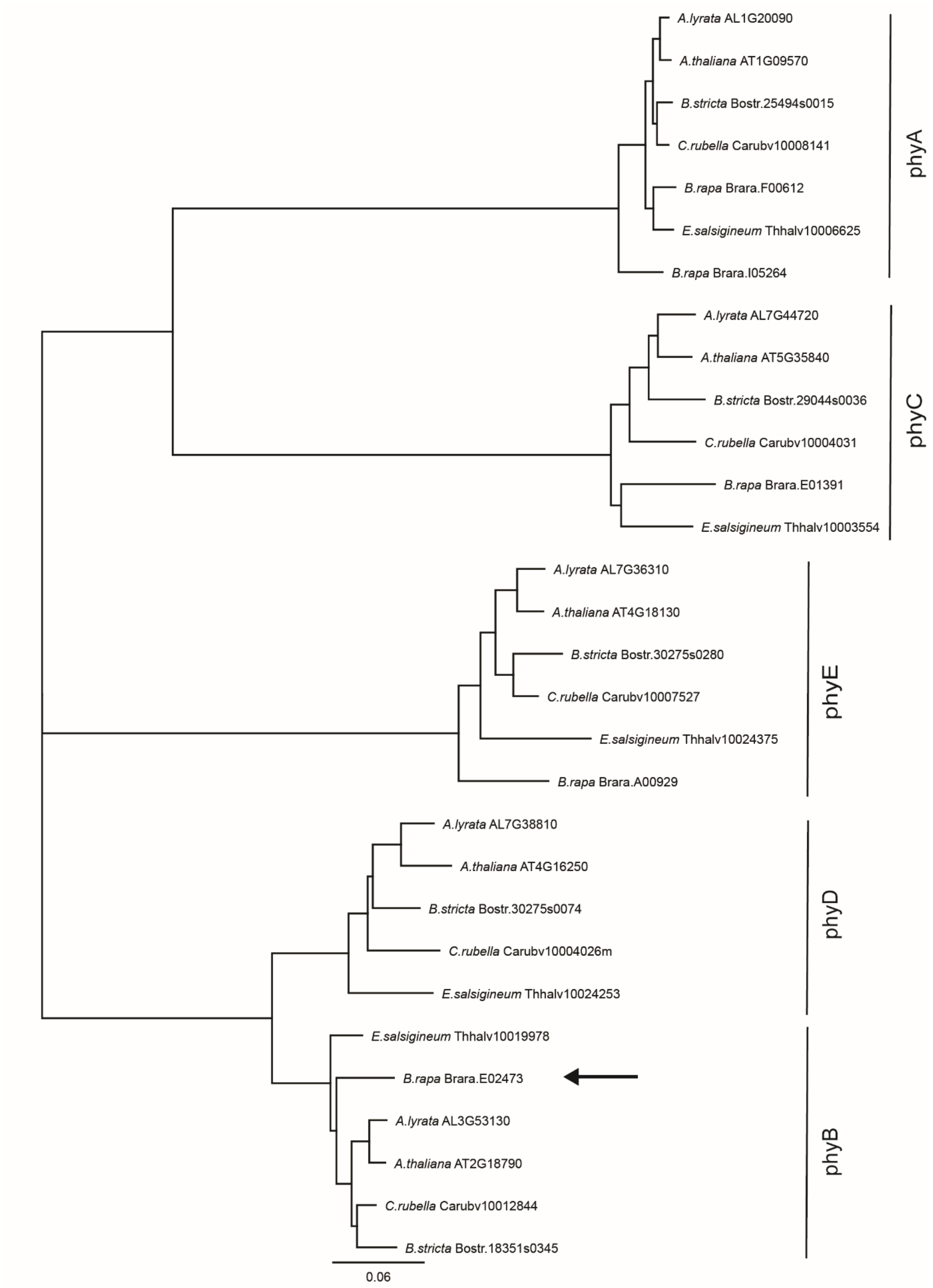

Supplemental Figure 3: Amino acid allignment of Phytochrome homologs in the *Brassicaceae*.

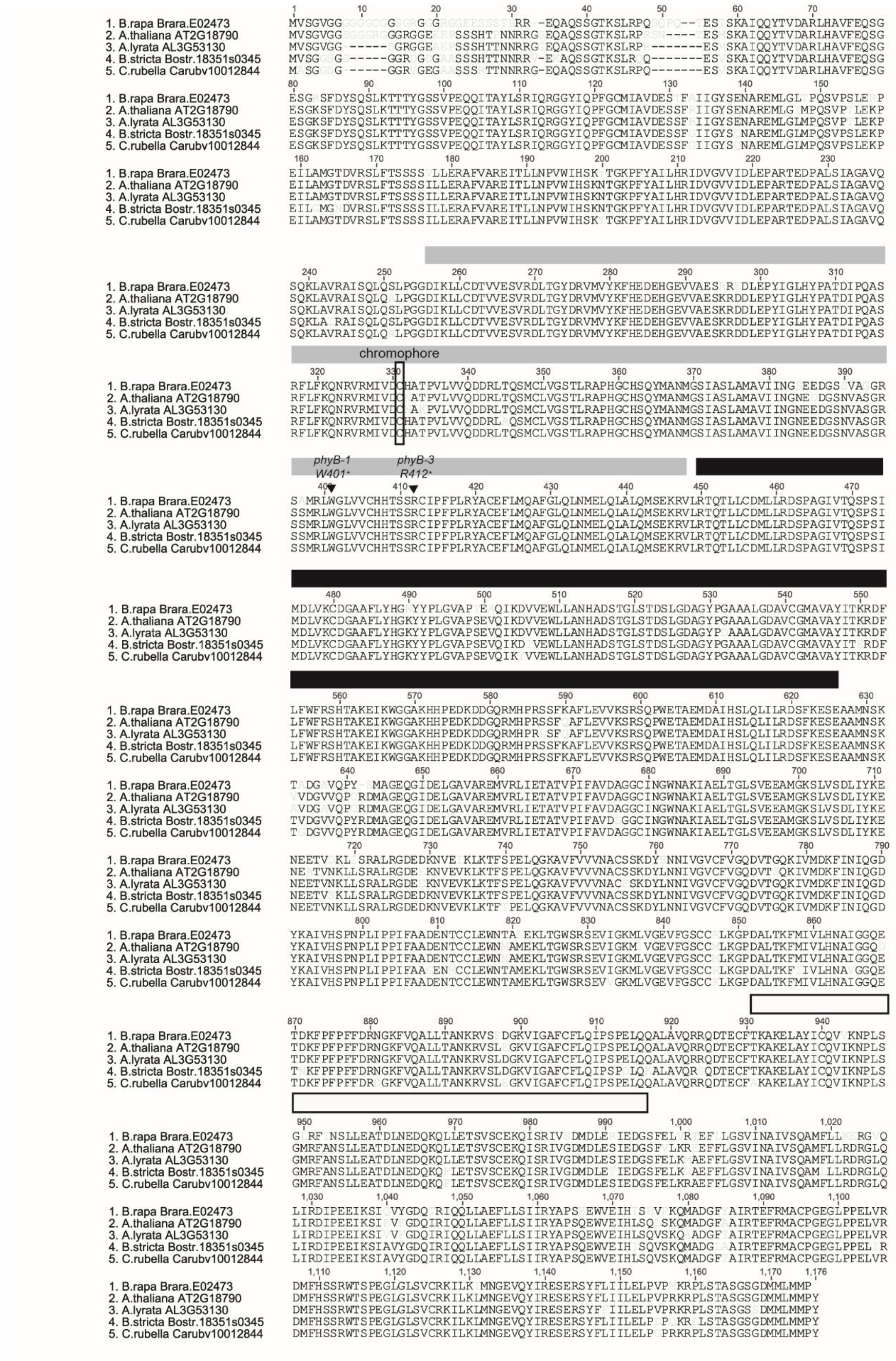

Supplemental Figure 4: Amino acid alignment of putative *Brasicaceae* phyB orthologs.

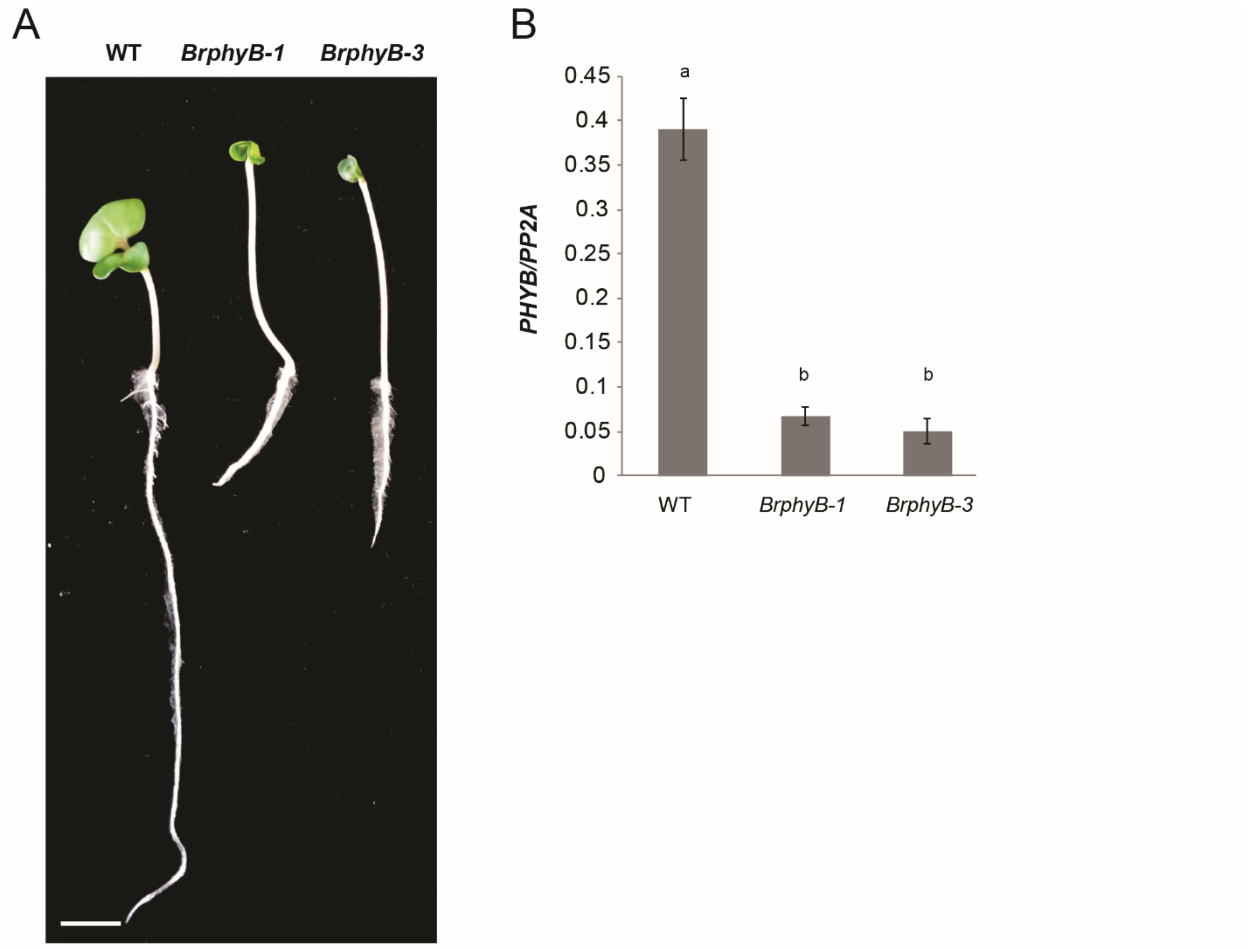

Supplementary Figure 5: Wild type and *BrphyB* mutant seedlings grown for 4 days under red light (A). *BrPhyB* expression in *B.rapa* mutants (B). Scale bar 1cm.

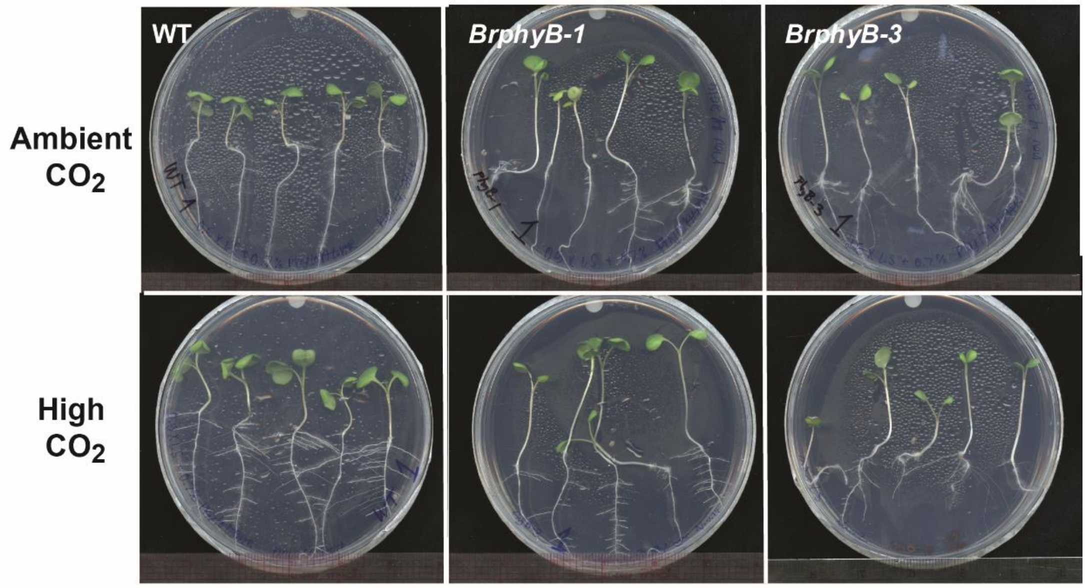

Supplemental Figure 6: Response to elevated CO_2 requires BrPhyB.

A

Plant	Time (min)	Photosynthesis (μmol $CO_2/m^2/s$)	Stomatal Conductance (mol/m^2/s)
1	5	21.292	0.926
1	10	21.828	0.836
1	15	22.600	0.779
2	5	17.252	0.374
2	10	23.226	0.367
2	15	23.298	0.364
3	5	20.627	0.813
3	10	22.429	0.767
3	15	23.305	0.740
4	5	15.992	0.648
4	10	18.763	0.620
4	15	19.693	0.608
5	5	19.222	0.534
5	10	22.072	0.586
5	15	23.657	0.646

B

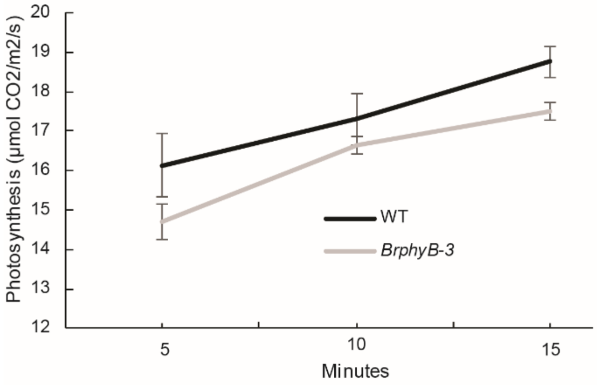

Supplemental Figure 7: Photosynthetic and stomatal conductance time course of 3 week old *B.rapa* wild-type plants. A, photosynthetic rate and stomatal conductance over 15 minutes of 3 week old plants at 1500 μmol/m2/s light, and 450ppm CO2. B, photosynthetic rate of 3 week old plants at 500 μmol/m2/s and 450ppm CO2, error bars are standard deviation.
